# Inferior mesenteric artery arteriovenous malformation, a rare cause of ischemic colitis: A case report

**DOI:** 10.1097/MD.0000000000033413

**Published:** 2023-03-24

**Authors:** Ching-Tang Chang, Wei-Xiong Lim, Ting-Ting Liu, Yueh-Ming Lin, Ching-Di Chang

**Affiliations:** a Department of Emergency, Kaohsiung Municipal Ta-Tung Hospital, Kaohsiung, Taiwan; b Department of Radiology, Kaohsiung Chang Gung Memorial Hospital, Chang Gung University College of Medicine, Kaohsiung, Taiwan; c Department of Pathology, Kaohsiung Chang Gung Memorial Hospital and Chang Gung University College of Medicine, Kaohsiung, Taiwan; d Department of Surgery, Division of Colorectal Surgery, Kaohsiung Chang Gung Memorial Hospital and Chang Gung University College of Medicine, Kaohsiung, Taiwan.

**Keywords:** arteriovenous malformation, inferior mesenteric artery, ischemic colitis

## Abstract

An arteriovenous malformation (AVM) is an abnormal tangle of blood vessels that connects the arteries and the veins. Because normal capillary bed is partially or completely absented in the AVM, the blood passes quickly from the arteries to the veins, which disrupts normal blood flow and oxygen supply to the surrounding tissues. This is called “steal phenomenon,” and in the inferior mesenteric artery (IMA) territory, this may lead to abdominal pain, gastrointestinal bleeding, portal hypertension, and even ischemic colitis.

**Patient concerns:**

A 67-year-old man presented to our emergency department because of left side abdominal pain.

**Diagnoses:**

The abdominal computed tomography with contrast enhancement revealed a cluster of abnormal vascular lesions abutting the IMA with early opacification of the left colonic marginal vein. In addition, poor enhancement of segmental colonic wall was found from proximal descending colon to middle rectum. The diagnosis of AVM of the IMA and ischemic colitis was made.

**Interventions:**

The patient underwent left hemicolectomy as well as the AVM resection.

**Outcomes:**

He was discharged uneventfully after the surgery without complications.

**Lessons:**

IMA AVM carries the risk of ischemic colitis. computed tomography scan is helpful not only to the diagnosis of AVMs but also to exclude other lesions as well. Treatment options include endovascular embolization, surgical intervention, and a combination of both. Due to the complexity of this disease, treatment requires a case-specific multidisciplinary approach and a coordination of medical, radiological, and surgical staffs.

## 1. Introduction

An arteriovenous malformation (AVM) is an abnormal tangle of blood vessels that connects the arteries and the veins. It can be either primary or secondary due to previous surgery or trauma. Because normal capillary bed is partially or completely absented in the AVM, the blood passes quickly from the arteries to the veins, which disrupts normal blood flow and oxygen supply to the surrounding tissues. This is called “steal phenomenon,” and in the inferior mesenteric artery (IMA) territory, this may lead to abdominal pain, gastrointestinal bleeding, portal hypertension, and even ischemic colitis.^[1–4]^ Treatment options include endovascular embolization, surgical intervention, and a combination of both. Due to the complexity of this disease, treatment requires a case-specific multidisciplinary approach and a coordination of medical, radiological, and surgical staffs.

## 2. Case report

A 67-year-old man presented to our emergency department because of left side abdominal pain for several days. There was no fever, vomiting, diarrhea, or specific past medical history.

On arrival, his vital signs were stable. The physical examination revealed let side abdominal tenderness. Laboratory data showed white blood cell count 8.0 (1000/uL), hemoglobin 11.4 (g/dL), and high C-reactive protein 151.8 (mg/L). The renal function, hepatic enzyme, pancreatic enzyme, and blood sugar were all within normal range.

**Figure 1.**
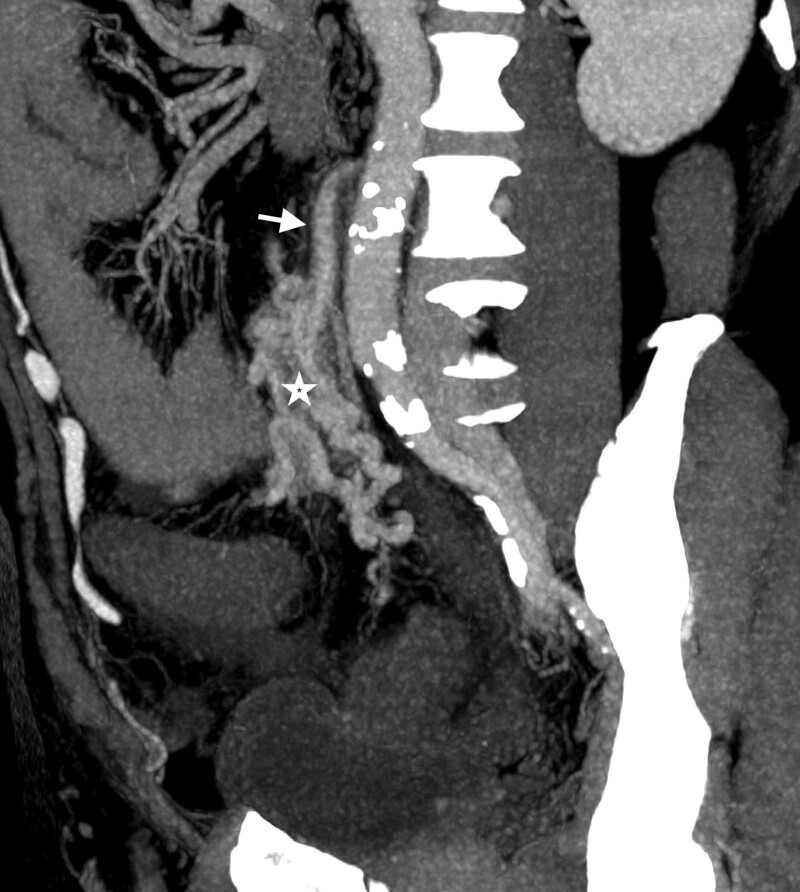
Maximum intensity projection on sagittal view demonstrated a cluster of abnormal early enhancing vascular nidus (asterisk) surrounding the engorged IMA (arrow). IMA = inferior mesenteric artery.

**Figure 2.**
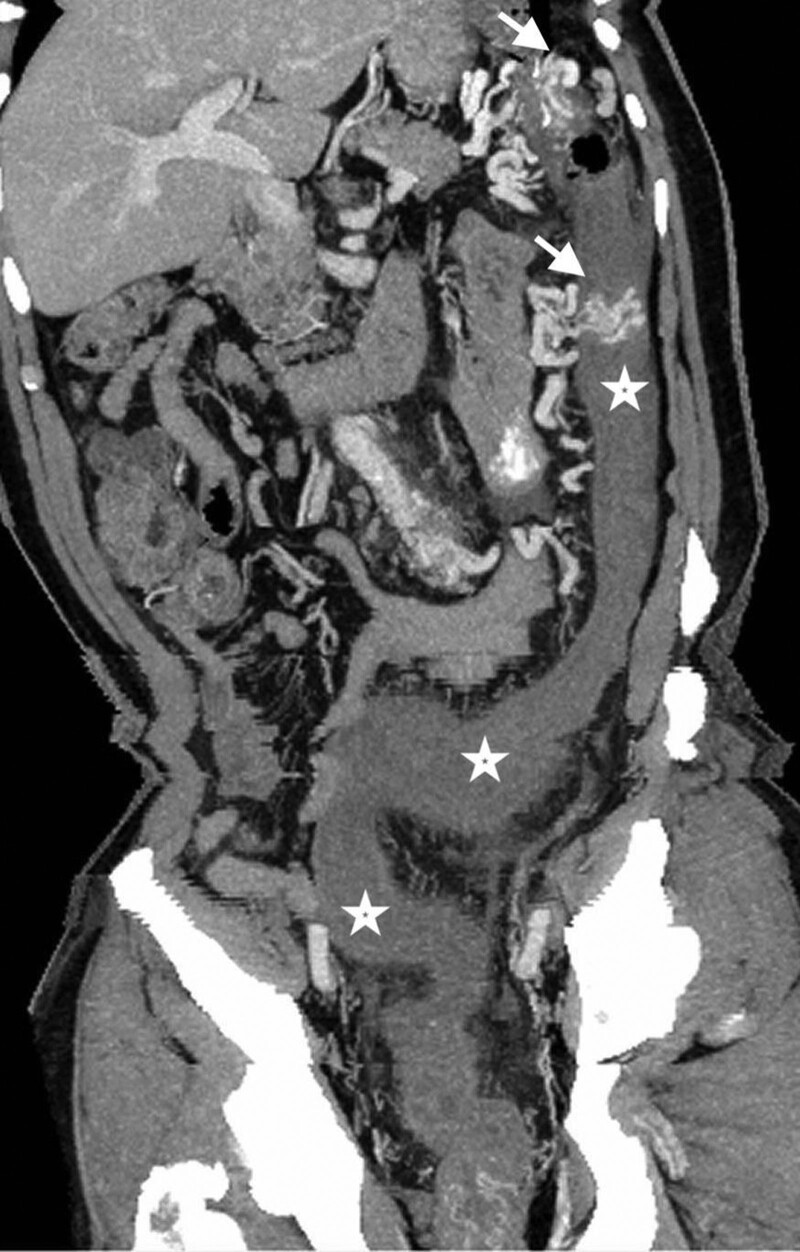
Curved planar reconstruction on coronal view showing the edematous colonic wall with poor enhancement extending downward from descending colon to middle rectum (asterisks). Two clusters of dilated venous structure in the proximal descending colon were shown (arrows).

**Figure 3.**
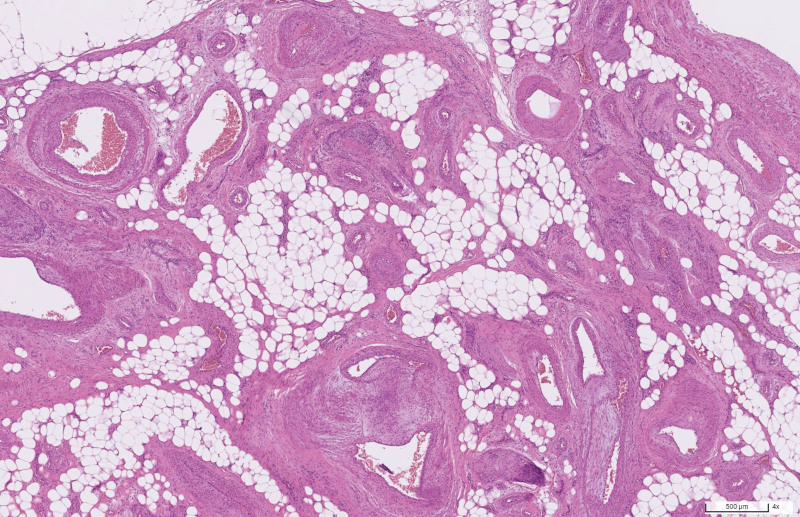
Histopathologic examination of the excised specimen. Mixture of arteries, veins, and small vessels with variably sized lumina separated by fibroadipose tissue consistent with AVM. The veins reveal hypertensive changes of intimal and mural thickening. [H&E stain; original magnification: ×40]. AVM = arteriovenous malformation.

Because intra-abdominal infection was highly suspected, the computed tomography (CT) was arranged. The abdominal CT with contrast enhancement revealed a cluster of abnormal vascular lesions abutting the inferior mesenteric artery with early opacification of the left colonic marginal vein (shown in Fig. 1). In addition, poor enhancement of segmental colonic wall was found from proximal descending colon to middle rectum (shown in Fig. 2). The diagnosis of arteriovenous malformation of the inferior mesenteric artery and ischemic colitis was made. The patient underwent left hemicolectomy as well as the AVM resection. The pathology report confirmed the diagnosis of AVM (shown in Fig. 3). He was discharged uneventfully after the surgery without complications.

## 3. Discussion

AVMs are aberrant vascular connections between the arteries and veins. The etiology of AVMs can be either primary due to developmental vascular defects or secondary due to previous surgery or trauma. AVMs can affect any organs, but they are mostly seen in the brain. Less frequently, the AVMs can involve the extremities, trunk, and viscera of the chest and abdomen. AVMs in the IMA territory are extremely rare.

Because normal capillary bed is partially or completely absented in the AVM, the blood passes quickly from the arteries to the veins, which disrupts normal blood flow and oxygen supply to the surrounding tissues. This is called “steal phenomenon” and the clinical presentations are usually flow dependent. When it comes to the IMA territory, reduced arterial blood flow and venous congestion may lead to abdominal pain, gastrointestinal bleeding, portal hypertension, and even ischemic colitis.^[5,6]^

Vascular imaging is usually required to confirm the diagnosis and to identify the extent of the lesion. The catheter-based angiography is helpful to determine the extent and severity of the AVMs. However, the catheter-based angiography lacks the ability to exclude other potential life-threatening diseases. As a result, the CT scan may be the modality of choice because it is helpful not only to the diagnosis of AVMs but also to exclude other intra-abdominal lesions as well. In this case, we also applied the curved planar reconstruction imaging technique to evaluate the gastrointestinal tract. The affected colon demonstrated edematous change, pericolic stranding, poor contrast enhancement, and venous congestion. These findings are suggestive of ischemic colitis caused by the AVMs and are also useful to guide the subsequent treatment of the disease.

The treatment of IMA AVMs requires a case-specific multidisciplinary approach because of its complexity. Treatment options include endovascular embolization, surgical intervention, and a combination of both.^[2,7–10]^ Embolization can be the definitive treatment alone or as a bridge prior to the operation. However, embolization carries the risk of bowel ischemia and recurrence of the AVMs had been reported if inadequate embolization was performed. Surgery is a potential curative treatment but bleeding is a major concern given the nature of the AVMs. Our patient underwent left hemicolectomy as well as the AVM resection without endovascular embolization because of lacking the experience of such endovascular intervention at our institution. Although we did not perform the endovascular embolization in advance, the surgery was done smoothly without complications and he was discharged uneventfully.

## 4. Conclusion

IMA AVM carries the risk of ischemic colitis. CT scan is helpful not only to the diagnosis of AVMs but also to exclude other lesions as well. Treatment options include endovascular embolization, surgical intervention, and a combination of both. Due to the complexity of this disease, treatment requires a case-specific multidisciplinary approach and a coordination of medical, radiological, and surgical staffs.

## Author contributions

**Writing – original draft:** Ching Tang Chang.

**Writing – review & editing:** Wei-Xiong Lim, Ting-Ting Liu, Yueh-Ming Lin, Ching-Di Chang.
